# Molecular Chiral Response Enhanced by Crosstalking Quasi-Bound States in the Continuum

**DOI:** 10.1021/acsphotonics.5c01225

**Published:** 2025-11-03

**Authors:** Diana Shakirova, Adrià Canós Valero, Daniil Riabov, Hatice Altug, Andrey Bogdanov, Thomas Weiss

**Affiliations:** † Institute of Physics, 27267 University of Graz, and NAWI Graz, Universitätsplatz 5, Graz 8010, Austria; ‡ 87254 Riga Technical University, Institute of Telecommunications, Riga 1048, Latvia; § Laboratory of Bionanophotonic Systems, Institute of Bioengineering, 27218 École Polytechnique Fédérale de Lausanne (EPFL), Lausanne 1015, Switzerland; ∥ Qingdao Innovation and Development Center of Harbin Engineering University, Qingdao 266500, China; ⊥ School of Physics and Engineering, ITMO University, 191002 St. Petersburg, Russia

**Keywords:** nanophotonics, chirality, sensing, resonant states, bound states in the continuum, dielectric metasurfaces

## Abstract

Identifying the handedness of chiral molecules is of fundamental importance in chemistry, biology, pharmacy, and medicine. Nanophotonic structures allow us to control light at the nanoscale and offer powerful tools for chiral sensing, enabling the detection of small analyte volumes and low molecular concentrations by harnessing optical resonances. Most existing strategies rely on intuitive concepts such as strong local field enhancement or large local optical chirality, often achieved by engineering electric and magnetic Mie resonances in dielectric or plasmonic nanostructures. Recent insights, however, reveal that the chiroptical response of resonant systems can be governed also by less obvious mechanisms such as modal crosstalk. In this work, we present a dielectric metasurface engineered to amplify the modal crosstalk by supporting two nearly degenerate, high-quality-factor resonant states known as quasi-bound states in the continuum. Our theoretical and numerical analysis predicts a pronounced differential transmittance that exceeds the detection threshold of standard spectrometers. In particular, the differential transmittance reaches up to 10^–2^ for the Pasteur parameter κ = 1 × 10^–4^. These findings advance the capabilities of nanophotonic sensors for chiral detection, paving the way toward ultrasensitive identification of molecular handedness in small volumes and concentrations within the experimentally detectable ranges.

## Graphical abstract

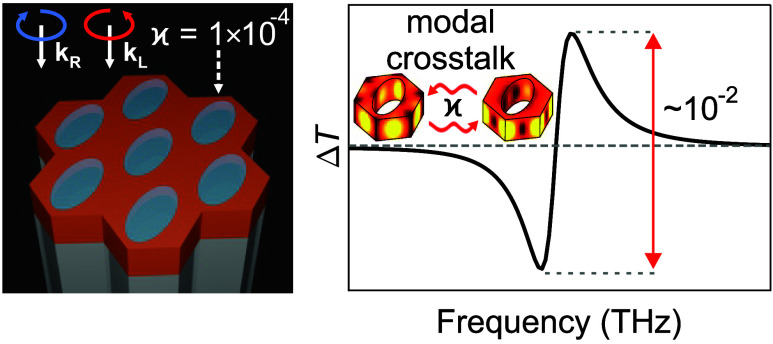

## Introduction

Chirality is known as the nonidentity of an object to its mirror image. This fundamental property is inherent in many organic molecules, chemical compounds, and drugs.^1–3^ Although chirality seems to be a purely geometrical characteristic, it crucially affects living organisms. The same molecules of opposite handedness (enantiomers) can act as a drug or as a toxin, respectively.^1^ Therefore, the detection of molecular handedness is extremely important in biology, chemistry, medicine, pharmacy, and food science.^4–9^

One of the most well-known approaches of enantiomeric discrimination is to measure circular dichroism (CD), the difference in absorbance of left- and right-handed circularly polarized (LCP and RCP) light by chiral molecules. Unfortunately, CD signals from natural chiral matter are very weak in the visible and infrared spectral ranges, so that they are below the detection limit for conventional CD spectroscopy in many practical applications, where only a limited number of molecules is available. The detection limit is typically around ≈10^–5^ in terms of absolute transmittance or absorbance difference.^10^ This is where nanophotonics comes into play. The use of resonances of plasmonic and dielectric nanostructures has been shown to significantly enhance chiral light-matter interaction and increase CD.^10–24^ These resonances are associated with the resonant states (or quasinormal modes) of the optical systems that can trap the light efficiently at certain incident frequencies.^25,26^ The quasinormal modes are characterized by complex eigenfrequencies, whose imaginary parts are associated with the total losses as a sum of radiative and absorption ones.^26,27^

One way to characterize chiral light-matter interaction involves evaluation of the so-called local optical chirality *C*.^28^ This quantity is proportional to the imaginary part of the scalar product between the electric and magnetic field, namely *C* ∼ Im­(**E** · H*),^28^ which is related to the absorbance difference between LCP and RCP light interacting with molecules at a certain position in space.^14,15,17,21,28,29^ Thus, *C* characterizes the degree of local field chirality, which motivated many studies of the chiral fields generated by *plasmonic* nanostructures in the first place.^10–17,29–31^ The plasmonic resonant states guarantee strong field enhancements within small volumes in the optical spectral range and may be used to achieve large *C* values. Both single particles^11–13^ and periodic arrays^10,14–17^ have demonstrated amplified chiral response from a sample placed in their vicinity. In particular, significant CD enhancement of the order of 10^3^ has been reported in^10^ for the case of a uniform chiral analyte placed at the edges of plasmonic nanorods. However, even this level of enhancement remains below the detection threshold of conventional spectrometers when only a limited analyte volume is available.

In recent years, *dielectric* nanophotonics has emerged as a promising alternative to plasmonics.^18–23,32–35^ Both theoretical^18–21^ and experimental^22^ works have shown the effect of CD enhancement on the order of 10–10^2^ by using all-dielectric or hybrid metal-dielectric platforms. The first reason for such efficiency is the ability to harness the magnetic resonances in dielectric structures, which in proper combination with the electric ones, can significantly increase the local optical chirality.^18–22,24,35,36^ The second reason is the long temporal confinement of light in dielectric materials. Quantitatively, the duration of light confinement by a resonant state can be characterized by the quality factor *Q*, which is the absolute value of the ratio of the real and twice the imaginary parts of its eigenfrequency ω_*n*_, *Q* = |Re­(ω_*n*_)/2Im­(ω_*n*_)|. Resonant states with significantly higher *Q* factors are accessible in dielectric nanoresonators due to smaller intrinsic material losses compared to plasmonic platforms.

Special cases of resonant states possessing infinite *Q* factors are bound states in the continuum (BICs). Originally proposed in quantum mechanics,^37^ the concept of BICs has been transferred to optics recently.^38,39^ However, the advantage of infinitely high *Q* factors of these resonant states cannot be used since BICs are uncoupled from free space plane waves. One way to overcome this obstacle is to involve deformation or structural imperfections, thus introducing radiative losses in a resonator and allowing for the plane waves outcoupling.^40^ In combination with intrinsic material losses, radiative losses limit the *Q* factors of the perturbed modes, which are referred to as so-called quasi-bound states in the continuum (quasi-BICs)—resonant states possessing finite, but still significantly high *Q* factors. Quasi-BICs have attracted significant attention in the nanophotonics community over the last decades and they have been used to demonstrate enhanced performance in lasing,^41,42^ active photonics,^43^ biophotonics,^44,45^ and polaritonics.^46^ In the chiral sensing, this concept has also been used for notable amplification of chiroptical response.^20,33,34,47^ In refs 33. and^34^, a huge enhancement of 10^3^–10^5^ order is stated for metasurfaces that support nearly degenerate quasi-BICs. Extremely high quality factors of resonant states with *Q* ≈ 10^4^–10^6^ are obtained for systems made of lossless dielectric material when introducing small geometrical perturbations. In this case, the coupling of the modes caused by chiral analyte is larger than the spectral distance between the corresponding eigenfrequencies, which allows the authors to operate in the strong-coupling regime. However, the suggested designs are challenging for experimental realization. First, they demand total film thickness control with the precision of less than 1 nm to ensure the strong-coupling regime. Moreover, the assumption of lossless material implies high crystallinity and absence of defects. Both requirements can be realized only using extremely expensive methods such as molecular beam epitaxy.^48^ Second, the suggested quality factors of the modes reaches up to *Q* ≈ 10^6^, which is hard to experimentally implement due to the induced scattering losses coming from fabrication imperfections.^49,50^ In addition, BICs have also been used to design metasurfaces exhibiting the high local optical chirality.^51–53^

In spite of this progress, only recently, a general electromagnetic theory of chiral light-matter interaction in arbitrary resonators has been developed.^27^ The idea behind this theory is the ability to reconstruct the optical response (i.e., transmission, reflection, and absorption) from a system via its resonant states.^25,26,54,55^ Moreover, it predicts the change in the optical response with a small perturbation introduced in the system, which is, in our case, given by the chiral analyte of interest. In the framework of this theory, this change can be disentangled into a sum of four contributions, namely, resonance shift, changes in the excitation and emission of resonances, and *modal crosstalk*. The resonance shift describes how strongly a particular resonance of an unperturbed structure spectrally shifts after inserting a chiral analyte. This contribution is present only in geometrically chiral structures,^27^ and has already been well studied.^14–16^ The second and third contributions are responsible for the change in optical response intensity between an unperturbed and perturbed resonator. This effect has also been realized in earlier works.^10,29^ The last mechanism, the modal crosstalk, implies any alteration of the optical spectrum caused by the interplay of spectrally close resonant states due to the presence of a chiral medium. While the first three processes have been widely realized, albeit without explicit recognition in most studies, the modal crosstalk for chiral sensing is rather unexploited. Although some works have combined electric and magnetic modes,^18,22,33–35^ so far, there was no strict recipe to maximize this contribution, which, in particular, allows us to benefit from a bisignate chiroptical response for the real-dominated Pasteur parameter, as shown further.

In this work, we investigate a theoretical framework to exploit the effect of the modal crosstalk for enhancing weak chiroptical signals. Notably, we uncover that the modal crosstalk can be significantly boosted by leveraging the interaction between two well-designed high-*Q* eigenmodes when they have opposite parity under inversion, and their resonant frequencies are nearly degenerate. In contrast with refs 33,34 ., we operate in the weak-coupling regime of the eigenmodes, allowing us to apply the electromagnetic theory of chiral light-matter interaction^27^ and rigorously explain the obtained spectral changes and CD enhancement. Our study not only sheds light on the known strategies to enhance CD, i.e., the use of collinear electric and magnetic fields, degeneracy, and high-*Q* modes, but also for the first time provides a robust theoretical explanation of the observed chiroptical response.

We start our exploration with the analysis of the expression for the modal crosstalk contribution derived in ref 27. and determine conditions for its maximization. Next, we propose a metasurface corresponding to the defined requirements and accounting for experimental restrictions, and confirm by both full-wave simulations (COMSOL Multiphysics^56^) and the modal theory the predicted CD enhancement. In addition to providing physical insights to explain the modal crosstalk, we achieve with our metasurface a differential transmittance on the order of 10^–2^ for κ = 1 × 10^–4^, which provides a promising route to beat the detection limit for small analyte volumes and low concentrations of chiral molecules. We also compare our metasurface to existing plasmonic^27^ and dielectric platforms,^18,22^ proving the benefits of our design.

## Results and Discussion

We start our analysis by formulating a theory of CD induced by a resonant structure. To efficiently characterize the interaction of incident light with a resonator, the optical scattering matrix *S* is widely used.^25,54,55,57^ In such planar systems, each element of the scattering matrix, *S*_**MN**_, represents the amplitude for light to scatter from an incoming channel **N** to an outgoing channel **M**, through either transmission or reflection. Here, we adopt the conventions from ref 27. and use the indices **N** and **M** as sets of quantum numbers that characterize a particular scattering channel. Each channel is defined by two parameters: the polarization of the light—left- or right-handed circularly polarized (L or R)—and the region of propagation—top (t) or bottom (b), denoting the superstrate or the substrate, respectively. For example, the absolute value squared of the matrix element |*S*_Rb,Lt_|^2^, which is also written as |*t*_RL_|^2^ or *T*_RL_, describes the transmittance of left-handed circularly polarized (LCP) light incident from the top into right-handed circularly polarized (RCP) light transmitted to the bottom. In periodic systems, **N** and **M** may also include diffraction orders, since scattering can occur into multiple diffraction channels. However, only the channels corresponding to the waves propagating in free space are considered. For arrays with subwavelength periodicity, only the zeroth diffraction order satisfies this condition. Since our study is limited to this regime, we omit the diffraction orders from the notation of the quantum numbers **N** and **M**.

If an arbitrary perturbation, which is a chiral analyte in our case, is inserted into a resonator, the scattering matrix changes from *S* to *S* + δ*S*, where δ*S* corresponds to the change upon the perturbation. This change can be calculated with a perturbation theory and expressed as a sum of the four terms associated with resonant effects: δ*S* = δ*S* ^ex^ + δ*S* ^em^ + δ*S* ^shift^ + δ*S* ^cross^.^27^ These contributions are the change in excitation δ*S* ^ex^ and emission δ*S* ^em^, defined by the coupling of modes with incoming and outgoing fields, respectively; the resonance shift δ*S* ^shift^, containing an overlap integral of the modes with themselves; and the modal crosstalk δ*S* ^cross^, which takes into account the interaction between different modes.

In general, CD is defined as the difference in absorbance Δ*A* for LCP and RCP light.^10,27,29,31,58^ The differential absorbance Δ*A* is related to the differential transmittance Δ*T* and the differential reflectance Δ*R*, which are the differences in transmittance and reflectance for LCP and RCP light, respectively, as CD = Δ*A* = −Δ*T* – Δ*R*. In special cases, such as a homogeneous medium or periodic arrays possessing symmetry *C*_3_ and higher,^58,59^ Δ*R* goes to zero. Therefore, one can associate CD with Δ*T* straightforwardly, which is also more feasible for experimental measurements.^17–19,22^ In this work, we propose a structure that demonstrates rather low differential reflectance, which allows us to use Δ*T* as a figure of merit (FOM). Furthermore, one should take into account circular polarization conversion (CPC) that appears for LCP and RCP light in structures possessing lower than *C*_3_ symmetries.^58,59^ Caused by elliptical birefringence, CPC can be assumed erroneously as CD.^58^ Therefore, we choose Δ*T* = *T*_LL_ – *T*_RR_ as a FOM to be maximized, with the assumption of CPC to be negligibly small, i.e., *T*_RL_, *T*_LR_ ≪ *T*_LL_, *T*_RR_, and *T*_RL_ – *T*_LR_ ≪ *T*_LL_ – *T*_RR_. Thus, the differential transmittance can be written as

$$\Delta T=\left|S_{\mathrm{Lb},\mathrm{Lt}}{|}^{2}-\right|S_{\mathrm{Rb},\mathrm{Rt}}{|}^{2}=|S_{\mathrm{Lb},\mathrm{Lt}}^{\left(0\right)}+\delta S_{\mathrm{Lb},\mathrm{Lt}}{|}^{2}-|S_{\mathrm{Rb},\mathrm{Rt}}^{\left(0\right)}+\delta S_{\mathrm{Rb},\mathrm{Rt}}{|}^{2}$$ 1

where *S* ^(0)^ and δ*S* are the unperturbed transmission amplitude and its perturbation correction, respectively.

We dedicate this work to the exploration of the perturbation in transmission amplitude dominated by the modal crosstalk, namely δ*S* ≈ δ*S* ^cross^, which is found as^27^

2 $$\delta S_{\mathrm{MN}}^{\mathrm{cross}}=\sum_{n\neq n\prime }\frac{a_{n,M}b_{n\prime ,N}\int_{V_{c}}i\omega c\kappa \left(E_{n}\cdot H_{n\prime }+H_{n}\cdot E_{n\prime }\right)dV}{\left(\omega -\omega_{n}\right)\left(\omega -\omega_{n\prime }\right)}$$

where *a*_*n*,**M**_ and *b*_*n*′,**N**_ are emission and excitation coefficients of the modes *n* and *n*′, respectively (see ref 27. for definition), ω and ω_*n*_ are, respectively, the angular frequency of the incident light and the eigenfrequency of the resonant state with index *n* (do not mix up with refractive index), *c* is the speed of light in vacuum, and **E**_*n*_ and **H**_*n*_ are electric and magnetic resonant field distributions. Finally, κ is the Pasteur parameter describing the chirality strength of an isotropic and homogeneous molecular solution.^10,29,58^ An opposite sign of κ corresponds to opposite handedness of the molecular solution.

The next step of our analysis involves establishing the prerequisites for Δ*T* to be maximized by the modal crosstalk δ*S* ^cross^. For this purpose, we consider a resonator supporting two resonant states with the indices *n* and *n*′ that are spectrally close to each other, i.e., the line widths of the corresponding resonances overlap. It is evident from eq 2 that degeneracy of the latter would guarantee a sharp drop of the denominator: Re­(ω_*n*_) = Re­(ω_*n*′_) and ω – Re­(ω_*n*_) ≈ 0 in the vicinity of the resonance. Therefore, the only nonzero contribution to the denominator is the product of Im­(ω_*n*_)­Im­(ω_*n*′_), which can be significantly reduced with the use of high-*Q* modes. Second, the choice of orthogonally polarized resonant states enhances the integrand in eq 2, namely, the scalar products of electric and magnetic modal fields **E**_*n*_·**H**_*n*′_ and **E**_*n*′_·**H**_*n*_ are maximized. Thus, spectral degeneracy of orthogonally polarized high-*Q* modes boost δ*S* ^cross^. To efficiently exploit the modes, one also has to maximize the frequency-dependent emission and excitation coefficients *a*_*n*,**M**_(ω) and *b*_*n*′,**N**_(ω), which represent the coupling of the modal fields to the outgoing and incoming waves, respectively.^27^ Therefore, *a*_*n*,**M**_ and *b*_*n*′,**N**_ can be enhanced by a proper choice of incoming and outgoing waves polarization, which increases the scalar product of the modal fields with the latter.

The final step of our analysis is to show how the established prerequisites can be realized in a realistic metasurface design. For this purpose, we propose a metasurface with a triangular unit cell containing a circular void. The metasurface is made of silicon nitride (Si_3_N_4_) and placed on a transparent substrate. We set the upper half-space to be water with *n*_H_2_O_ = 1.33, which is not shown in Figure 1(a) for illustrative reasons. This configuration resembles a racemic mixture of chiral molecules so that any spectral shift of the bare metasurface modes only originates in the deviation from this racemic mixture. Moreover, the water on the upper half-space ensures impedance matching with the substrate, which improves the quality factor of the involved resonances and, therefore, enhances the modal crosstalk.

**Figure 1:**
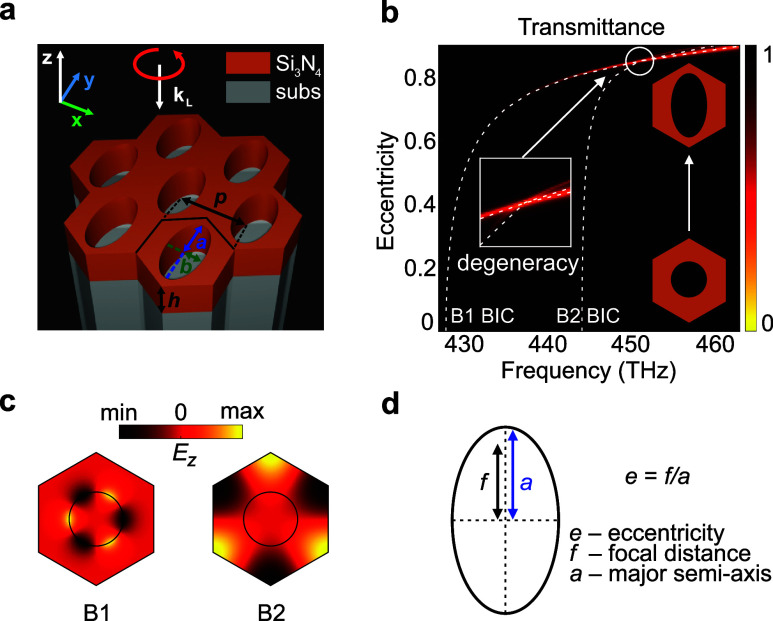
(a) Metasurface design as a periodic array of silicon nitride Si_3_N_4_ slab with triangular unit cells and refractive index *n*_Si_3_N_4__ = 1.9464 + 0.0012*i* on a model substrate with *n*_subs_ = 1.4. The unit cell with period *p* = 470 nm and height *h* = 230 nm contains elliptical void characterized by *a* = 208.5 nm and *b* = 110 nm as large and small semiaxes, respectively. (b) Transmittance map for the metasurface illuminated by left-handed circularly polarized light. Transmittance is calculated by full-wave simulations as a function of frequency and eccentricity, which is defined in panel (d). Dashed white lines represent the dispersion of the considered quasi-bound states in the continuum B1 and B2 calculated by eigenvalue solver. For *e* ≈ 0.85, a degeneracy in real parts of quasi-bound states in the continuum eigenfrequencies is observed. (c) Distribution of *E*_*z*_ as the *z* component of the electric field in the undeformed unit cell for B1 and B2. (d) Definition of the eccentricity: The initially circle-shaped cross section of the unit cell void is deformed by stretching along the *y* axis. The eccentricity is defined as *e* = *f*/*a* with *f* and *a* being the focal distance and the major semiaxis of the ellipse, respectively.

The system exhibits *C*_6*v*_ symmetry at normal incidence and supports symmetry-protected BICs.^60^ In particular, the metasurface demonstrates two BICs at 428 THz (≈701 nm) and 444 THz (≈676 nm) that are associated with the B1 and B2 irreducible representations, respectively^60^ [Figure 1(c)]. The modes transforming under these representations possess opposite parity under inversion and guarantee electric fields of the modes to be orthogonal to each other, aligning with our aim. Theoretically, the BICs exhibit an infinite *Q* factor,^37–39^ which also makes them perfect candidates for Δ*T* boosting. Further, we break the symmetry from *C*_6*v*_ to *C*_2*v*_ by stretching the circular void to an elliptical one as depicted in Figure 1(b). With such deformation, we perturb the resonant states and couple them to free space, transforming BICs to quasi-BICs.^38,39,61^ The deformation strength can be characterized by the hole eccentricity *e*, introduced in Figure 1(d). Additionally, this deformation allows us to tune the dispersion of quasi-BICs. Figure 1(b) shows a color map for the transmittance as a function of frequency and eccentricity. White dashed lines represent the dispersion of B1 and B2 BICs. Illumination under normal incidence with LCP light allows us to excite both orthogonally linearly polarized modes simultaneously.

We keep notations B1 and B2 throughout this study to refer to the corresponding quasi-BICs, since the *C*_2*v*_ group still contains the B1 and B2 irreducible representations. Moreover, the modes do not couple with each other for the chosen perturbation. The degeneracy in real parts of their eigenfrequencies is observed for the eccentricity *e* ≈ 0.85. The metasurface geometrical parameters corresponding to the modes’ degeneracy are depicted in Figure 1(a) and specified in the caption. To calculate the transmittance dependence on the eccentricity, only the major semiaxis *a* is varied, while all other parameters are fixed.

Once the proper parameters of the metasurface are chosen, we add a chiral analyte in the system. Here, we introduce a chiral analyte with the Pasteur parameter κ = 1 × 10^–4^, which is the upper limit of realistic values used in literature.^22,27^ In the Supporting Information, we show that for the chiral analyte with Re­(κ) ≫ Im­(κ), which is a typical case in the visible frequency range,^10,18,22,27^ the imaginary part of the Pasteur parameter plays a crucial role only for the bare chiral analyte and the corresponding enhancement factor, while negligibly affects the absolute chiroptical response induced by the metasurface. Therefore, we fix the real-valued Pasteur parameter, which allows us to use a sparser mesh to reduce the computational cost of the calculations. The additional degree of freedom is the amount of chiral solution to be used. On the one hand, enough space should be occupied by the chiral medium to use all the near fields provided by the metasurface for maximizing the numerator in eq 2. On the other hand, if there is too much chiral analyte, the chiroptical response becomes dominated not by the resonator enhancement but by the extra amount of molecular solution instead. To account for these aspects, the chiral sample is assumed to be only inside the elliptical voids and completely filling them [inset in Figure 2(a)]. We note that such a configuration implies a very small volume of the sample to be considered, which corresponds to the minimal enhancement provided by the metasurface. A detailed discussion on the optimal amount of the solution to be used is provided in the Supporting Information (Figure S4). Experimentally, a very similar setup can be implemented with surface functionalization.^62,63^ The surface of the structure should then be modified with capture probes, which ensure specific binding of the molecules of interest.

**Figure 2:**
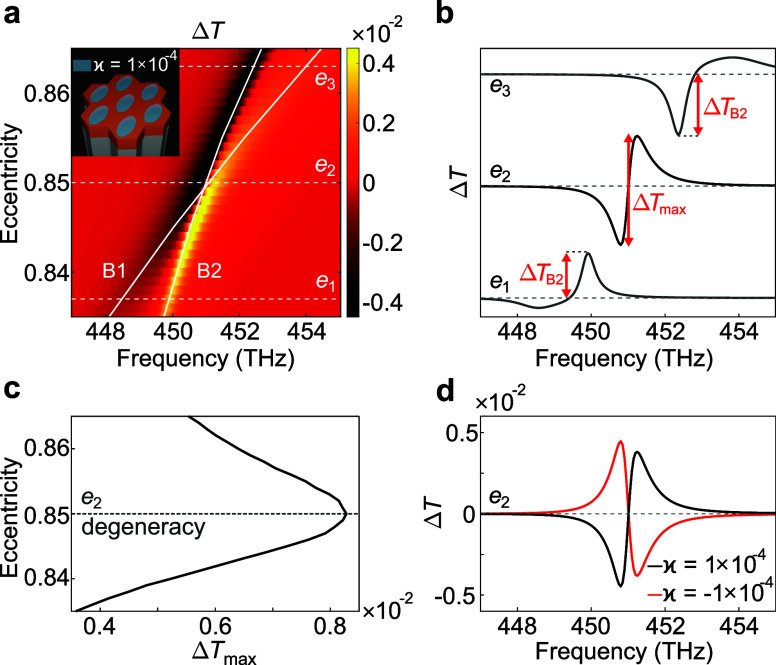
Δ*T* response of a chiral analyte placed on the metasurface. (a) Colormap for Δ*T* as a function of incident frequency and eccentricity. Solid white lines show the dispersion of B1 and B2 modes. Dashed lines highlight the eccentricity values to be discussed, and *e*_2_ ≈ 0.85, in particular, corresponds to the degeneracy. The inset sketches the metasurface with the chiral analyte placed into the voids. The Pasteur parameter here is κ = 1 × 10^–4^. (b) Examples of Δ*T* for *e*_1_ ≈ 0.84, *e*_2_, and *e*_3_ ≈ 0.86. It is seen that the amplitude of the bisignate response Δ*T*_max_ observed at the degeneracy at *e*_2_ significantly exceeds the chiral responses associated with single quasi-BICs located further from the degeneracy at *e*_1_ and *e*_3_. (c) Amplitude Δ*T*_max_ as a function of eccentricity. The highest value corresponds to the degeneracy at *e*_2_. (d) Δ*T* calculated for two Pasteur parameter values with opposite signs. Sign switch of the spectrum guarantees that Δ*T* is provided by the chiral nature of the analyte, proving the applicability of the system for handedness discrimination.

The chiroptical response of the system in the vicinity of the degeneracy is plotted as a function of frequency and eccentricity in Figure 2(a). Here, the solid white lines show the dispersion of the quasi-BICs, and the color represents Δ*T*. To prove the advantage of the degenerate configuration, we investigate Δ*T* at the eccentricities below, above and in close proximity to the degeneracy. In Figure 2(b), Δ*T* spectra corresponding to the eccentricity values *e*_1_ ≈ 0.84, *e*_2_ ≈ 0.85, and *e*_3_ ≈ 0.86 are shown. It is clearly seen that the case of degenerate resonant states allows us to make use of the bisignate signal shape, which is originally known as bisignate Cotton effect in supramolecular systems,^3^ and partially discussed in^32,64^ by Droulias and Bougas. We label the peak to peak signal amplitude as Δ*T*_max_, which significantly exceeds |Δ*T*_B2_| provided by the single quasi-BIC B2 demonstrated at *e*_1_ and *e*_3_. One can measure not only the single peak amplitude at *e*_1_ and *e*_3_, but also the peak-to-peak value Δ*T*_max_. Figure 2(c) shows the dependence of Δ*T*_max_ on the eccentricity. We flipped the axes to keep them consistent with Figure 2(a). The results indicate that the highest Δ*T*_max_ is achieved for the degenerate quasi-BICs. To make sure that the chiroptical response of the system is defined by intrinsic chirality of the analyte, we calculate Δ*T* for opposite signs of the Pasteur parameter. Indeed, Δ*T* flips its sign, as shown in Figure 2(d).

Finally, we investigate that the modal crosstalk is the main mechanism of Δ*T* enhancement. The contributions from δ*S* ^ex^, δ*S* ^em^, δ*S* ^shift^, and δ*S* ^cross^ can be calculated straightforwardly from the modal fields according to the theory explained in ref 27. Figure 3(a) displays the results of the modal analysis. The upper plot shows the comparison between the modal theory and numerical full-wave calculations. Notably, both approaches are in good agreement, despite the fact that only two modes B1 and B2 are used for the modal theory. The small deviation is caused by the background contributions of other modes located outside the considered spectral range. The lower panel depicts the contributions of the individual terms. It is clearly seen that the modal crosstalk, schematically shown in Figure 3(b), unambiguously dominates over the others.

**Figure 3:**
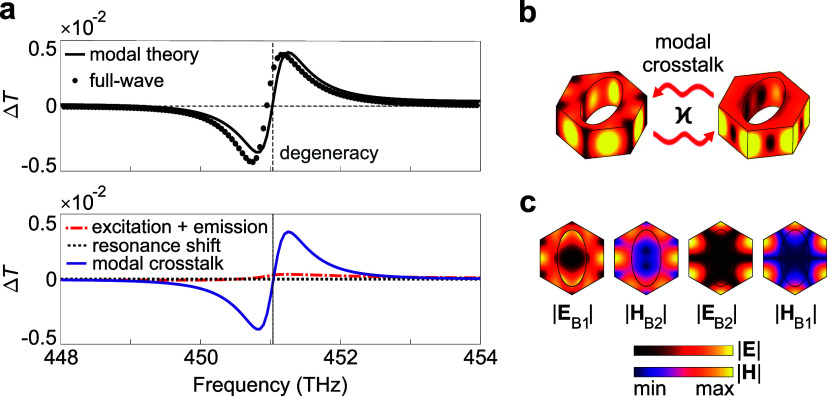
Modal theory. (a) Modal theory calculations. The upper plot shows Δ*T* predicted by modal theory (solid line) and full-wave calculation (dots). The results demonstrate good agreement. In the lower plot, all mechanisms contributing to the Δ*T* are presented separately. As was expected, the dominant mechanism is the modal crosstalk defined in eq 2. (b) Scheme of the modal crosstalk between the considered quasi-BICs, which is the main mechanism contributing to the enhancement of the chiral response. (c) Field distribution in the unit cell for B1 and B2 modes. Two colormaps are associated with absolute values of electric and magnetic fields. It is clearly seen that the electric and magnetic fields of the different modes possess the same symmetry.

In addition, Figure 3(c) shows the field distributions for the two modes at the degeneracy. It can be seen that the electric field of B1 has the same distribution pattern as the magnetic field of B2. The same holds for the magnetic field of B1 and the electric field of B2. This fact ensures to maximize the numerator in eq 2.

In order to demonstrate the potential of our approach, we compare our results to several key works in chiral sensing.^18,22,27^ There exists a large body of literature claiming significant CD enhancements in complex designs, which have so far not been experimentally validated.^13,20,21,29,34^ To ensure a meaningful comparison, we have deliberately chosen to benchmark our results only against well-established designs and experimentally confirmed studies.

In refs 18,22 ., the authors presented metasurface designs supporting the combination of electric and magnetic dipoles. To obtain comparable values, we have transformed their results to the differential transmittance and estimated the corresponding enhancement to be

3 $$\Delta T_{\mathrm{enh}}=\frac{\Delta T}{\Delta T_{\mathrm{ch}}}$$

where Δ*T*_ch_ is the differential transmittance of the bare chiral analyte with the Pasteur parameter κ = (1 + 0.01*i*) × 10^–4^, which is used in refs 18,22 . For each platform, we performed two simulations to estimate Δ*T*_enh_. In the first simulation, we considered the chiral analyte spatially distributed in the unit cell as stated in each reference, but the metasurface was removed to obtain Δ*T*_ch_. In the second simulation, we add the metasurface and calculate Δ*T*. This approach allows us to remove significant dependence of the results on the volume of chiral analyte. For all designs, we take the highest enhancement obtained, including peak-to-peak amplitude for those that present bisignate spectra. We show that the triangular metasurface provides 10^2^–10^3^ higher enhancement compared to these works.

We also compare our results with a prototypical periodic array of plasmonic nanorods, studied in refs 10,27 . For κ = (1 + 0.01*i*) × 10^–4^, Both et al. obtain the differential absorbance enhancement Δ*A*_enh_ ≈ 350, while the same quantity achieved with the triangular metasurface is 1000-fold. The results of the described comparison are summarized in Table 1.

**Table 1:** Literature Comparison^a^

reference	FOM	enhancement
Mohammadi et al.	Δ*T*_enh_	4.2
García-Guirado et al.	Δ*T*_enh_	50
Both et al.	Δ*A*_enh_	350
this work	Δ*T*_enh_	1950
	Δ*A*_enh_	1000

a FOMs from key works in chiral sensing are transformed to Δ*T*_enh_ (Δ*A*_enh_) and compared to the triangular metasurface.

## Conclusion

We have presented a metasurface design for molecular chiral sensing based on nearly degenerate quasi-BICs. The main mechanism driving the chiroptical response in our work is the modal crosstalk, and to maximize it, we have theoretically established a general strategy and confirmed by the modal theory and full-wave simulations. We have shown that in the special case of the degenerate, orthogonally polarized quasi-BICs possessing opposite parity, the modal crosstalk mechanism becomes dominant and significantly boosts the differential transmittance of the system.

We have also compared our metasurface to experimentally demonstrated^22^ and well-established designs^18,27^ in key works of chiral sensing, and unambiguously surpassed the previous results. It has been shown that our design provides significant enhancement of the FOM without requiring any signal postprocessing. We believe that our work paves the way to an experimentally feasible approach in chiral sensing that will allow for reliable handedness discrimination of small analyte volumes and low concentrations.

## Supplementary Material



## Notes

Methods, transmittance spectra for the real and complex Pasteur parameter, transmittance spectra for different eccentricities, and discussion on the amount of the chiral analyte to be used (PDF)
