# Supplementary material for: Molecular Chiral Response Enhanced by Crosstalking Quasi-Bound States in the Continuum
Source: ACS Photonics. 2025 Nov 3;12(11):6011–8. doi: 10.1021/acsphotonics.5c01225 (PMC12636083; doi:10.1021/acsphotonics.5c01225)
Supplement: Supplementary file 1 [file ph5c01225_si_001.pdf]

Supporting Information for Molecular chiral response enhanced by crosstalking quasi-bound states in the continuum

Diana Shakirova,† Adrià Canós Valero,†,‡ Daniil Riabov,¶ Hatice Altug,¶ Andrey Bogdanov,§,‖ and Thomas Weiss*,†

†*Institute of Physics, University of Graz, and NAWI Graz, Universitätsplatz 5, Graz 8010, Austria*

‡*Riga Technical University, Institute of Telecommunications, Riga, 1048, Latvia*

¶*Laboratory of Bionanophotonic Systems, Institute of Bioengineering, École Polytechnique Fédérale de Lausanne (EPFL), Lausanne 1015, Switzerland*

§*Qingdao Innovation and Development Center of Harbin Engineering University, Qingdao, 266500, China*

‖*School of Physics and Engineering, ITMO University, 191002, St. Petersburg, Russia*

E-mail: thomas.weiss@uni-graz.at

10 pages, 4 figures

Methods

Assuming a homogeneous and isotropic medium, the Pasteur parameter κ can be taken into account by the chiral constitutive equations (in Gaussian units):[1]

$$\begin{aligned} \mathbf{D} &= \varepsilon\mathbf{E} - i\kappa\mathbf{H} \\ \mathbf{B} &= \mu\mathbf{H} + i\kappa\mathbf{E}, \end{aligned} \tag{S1}$$

where ε is dielectric permittivity, μ is permeability, $\mathbf{D}$ is electric displacement, $\mathbf{B}$ is magnetic induction, and $\mathbf{E}$ and $\mathbf{H}$ are electric and magnetic fields, respectively. These equations can be realized in COMSOL Multiphysics full-wave simulation[2] that is used to calculate ΔT of the metasurface with a chiral analyte. The mirror symmetry mesh,[3] additionally combined with the swept mesh along the direction normal to the triangular metasurface (TMS), is applied to get rid of the numerical differential transmittance.

In this work, we consider the Pasteur parameter $\kappa = 1 \times 10^{-4}$, which is the upper limit for realistic values.[4,5] In general, the Pasteur parameter can also be a complex number $\kappa = (1 + 0.01i) \times 10^{-4}$ with the imaginary part usually being two orders smaller than the real one in the visible frequency range.[4–6] Hence, to resolve physical effects of this smaller imaginary part, an approximately two orders of magnitude finer mesh is required. However, we show in Fig. S1(a) that the imaginary part does not significantly affect ΔT of the system in this case, but rather the chiroptical response of the bare chiral analyte discussed in the next section. We also exploit the linear dependence of ΔT on the Pasteur parameter κ,[5,7] which implies $\Delta T(\kappa = 1 \times 10^{-4}) = \Delta T(\kappa = 1 \times 10^{-3})/10$ [Fig. S1(b)]. Therefore, we use $\kappa = 1 \times 10^{-3}$ in the simulations with the subsequent scaling of ΔT by a factor of 10. The choice of the purely real Pasteur parameter with scaled ΔT allows us to use a sparser mesh, keeping the computational cost lower without loss of physics.

In general, the differential transmittance is defined as $\Delta T = T_{\mathrm{LL}} + T_{\mathrm{RL}} - T_{\mathrm{RR}} - T_{\mathrm{LR}}$, taking into account the polarization conversion terms T_{RL} and T_{LR}. The latter appear in

structures possessing symmetry lower than C_3,[8,9] but we verify that polarization conversion for the TMS is negligible $T_{\mathrm{RL}} - T_{\mathrm{LR}} \ll T_{\mathrm{LL}} - T_{\mathrm{RR}}$, and $\Delta T \approx T_{\mathrm{LL}} - T_{\mathrm{RR}}$ [Fig. S1(c)]. This approximation is used throughout the work. The differential reflectance arises in the considered structure for the same symmetry reason,[8,9] which in general can compensate the differential transmittance due to the opposite sign and reduce the CD signal. We show in Fig. S1(d) that there is indeed non-zero differential reflectance $\Delta R \neq 0$, which is not large enough to suppress ΔA, though. Since the latter stays easily detectable, we can still use ΔT as a figure of merit.

Real and complex Pasteur parameter

In general, the Pasteur parameter is a complex value, where the real part $\mathrm{Re}\{\kappa\}$ is responsible for optical rotatory dispersion, and the imaginary part $\mathrm{Im}\{\kappa\}$ corresponds to CD in natural chiral molecules.[5,10,11] However, it has been shown that in nanophotonic systems, $\mathrm{Re}\{\kappa\}$ also contributes to CD, or alternatively ΔT,[5,7] due to the coupling between a nanoresonator and a chiral analyte. Furthermore, if the real part is dominant, i.e., $\mathrm{Re}\{\kappa\} \gg \mathrm{Im}\{\kappa\}$, which is a typical case in the visible frequency range, ΔT is fully dominated by $\mathrm{Re}\{\kappa\}$ due to the linear dependence of the chiroptical response on κ.[5,7] The latter is also confirmed by the full-wave simulations in Fig. S1(a). Therefore, the use of the purely real Pasteur parameter is valid for estimation of ΔT of the TMS with the inserted chiral analyte in absolute units. Nevertheless, the role of the imaginary part $\mathrm{Im}\{\kappa\}$ stays crucial for the differential transmittance of the bare chiral analyte ΔT_{ch} uncoupled from the resonator. We demonstrate the latter in Fig. S2(a,b), where ΔT_{ch} induced by the purely real Pasteur parameter is on the order of 10^{-11}, which is comparable to the noise signal. Notably, the introduction of even a minor imaginary part $\mathrm{Im}\{\kappa\} \ll \mathrm{Re}\{\kappa\}$ leads to the significant growth of ΔT_{ch} by 5 orders of magnitude, which corresponds to the physically meaningful response. This should be taken into account in the discussion of the enhancement factor defined in Eq. (3). Normalization of ΔT of the system

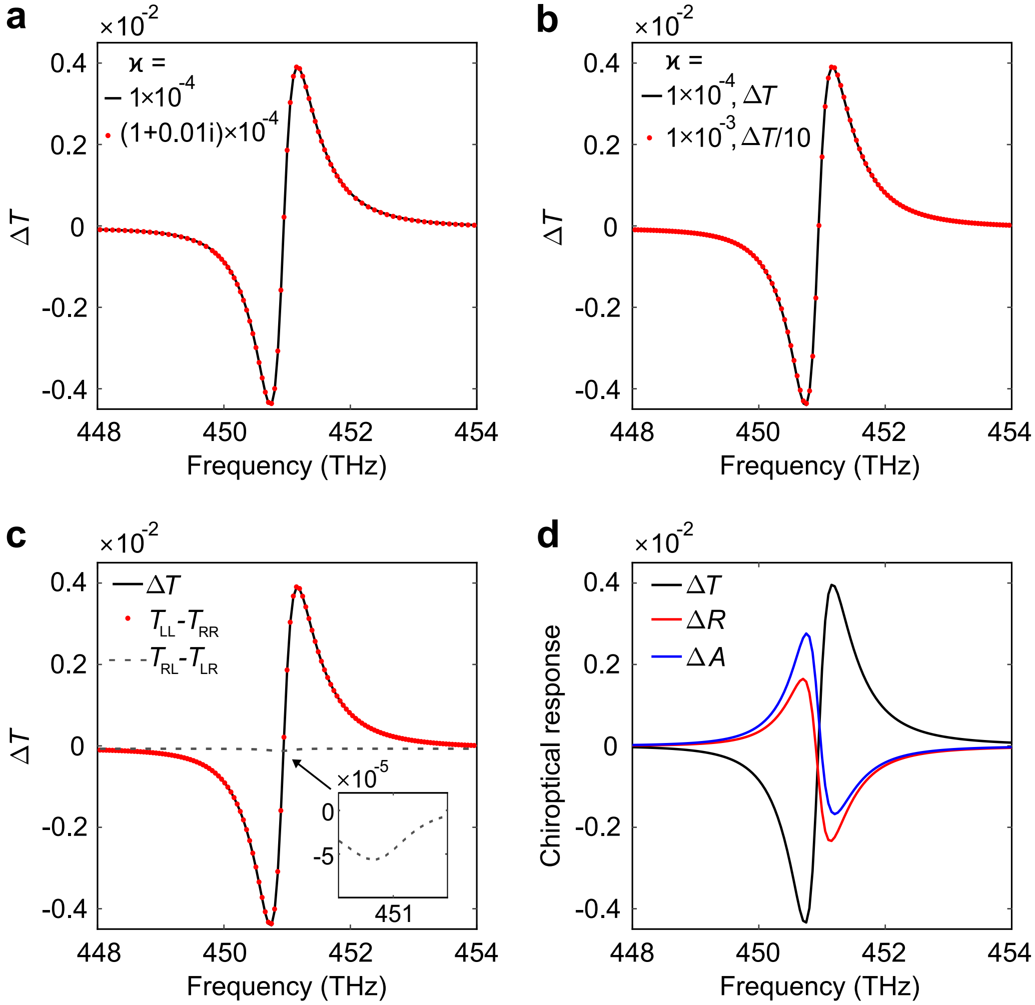

Figure S1: (a) ΔT of the TMS with the inserted real $\kappa = 1 \times 10^{-4}$ and complex $\kappa = (1 + 0.01i) \times 10^{-4}$ Pasteur parameter. (b) Linear scaling of the differential transmittance with the Pasteur parameter $\Delta T(\kappa = 1 \times 10^{-4}) = \Delta T(\kappa = 1 \times 10^{-3})/10$. (c) Contribution of polarization conversion $T_{\mathrm{RL}} - T_{\mathrm{LR}}$ to the total chiroptical response. (d) Chiroptical response of the TMS in terms of ΔT, ΔR, and ΔA, where $\Delta A = -\Delta T - \Delta R$. Non-zero ΔR does not suppress ΔA, which allows us to use ΔT as a figure of merit.

by the chiroptical response of the bare sample possessing the purely real Pasteur parameter provides an enhancement of seven orders of magnitude, which is impressive, but nonphysical [Fig. S2(c)]. Therefore, we estimate the enhancement provided by the TMS using $\Delta T_{\rm ch}$ from the chiral analyte with $\kappa = (1 + 0.01i) \times 10^{-4}$, observing peak-to-peak amplification of $\Delta T_{\rm enh} \approx 2000$ [Fig. S2(d)].

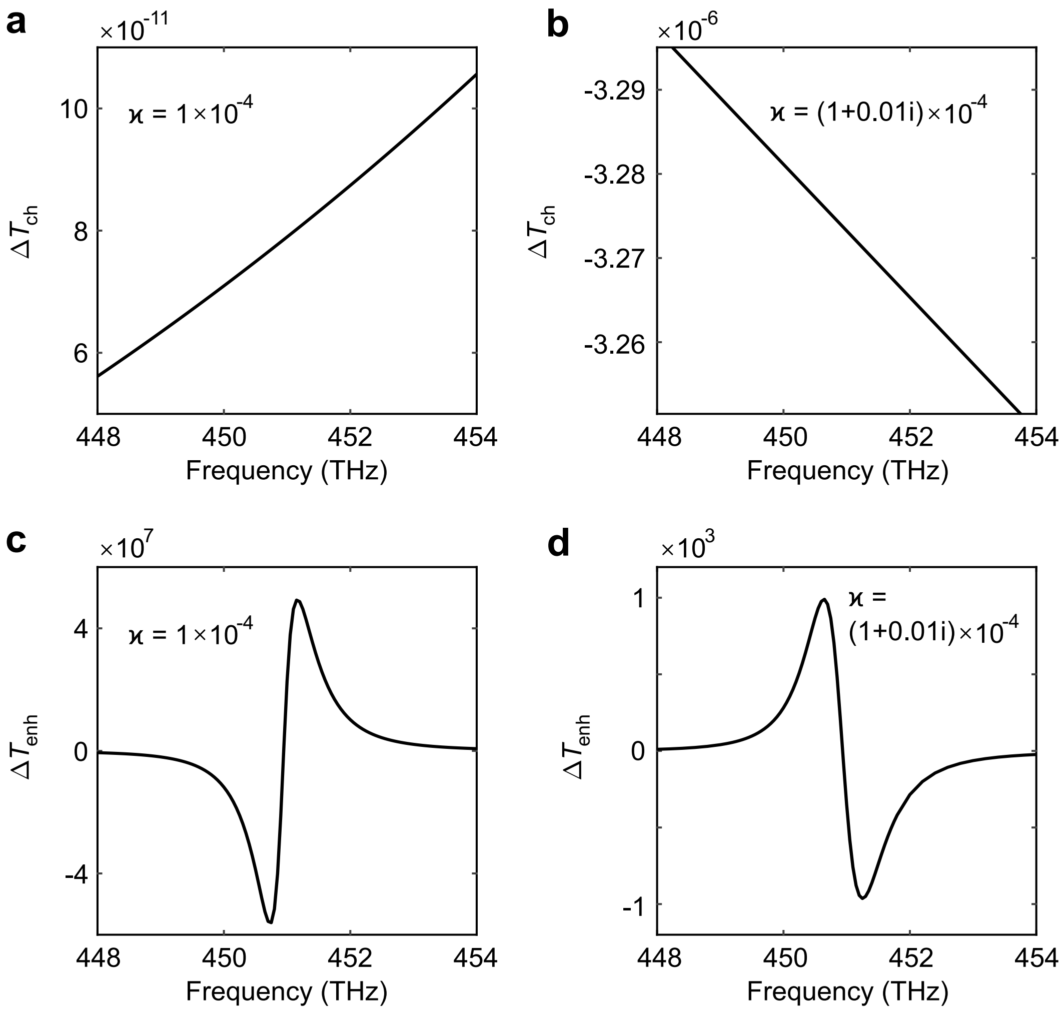

Figure S2: Influence of the imaginary part of the Pasteur parameter κ on the enhancement factor, demonstrated as the differential transmittance of the bare chiral analyte with the purely real (a) and complex (b) Pasteur parameter. The corresponding enhancement $\Delta T/\Delta T_{\rm ch}$ for the purely real and complex Pasteur parameter are displayed in panels (c) and (d), respectively.

TMS transmittance and relative differential transmittance

To estimate how strong the induced chiroptical response is for different eccentricities discussed in the main article, we provide LCP-transmittance spectra of the bare TMS as well as ΔT normalized by the latter in Fig. S3.

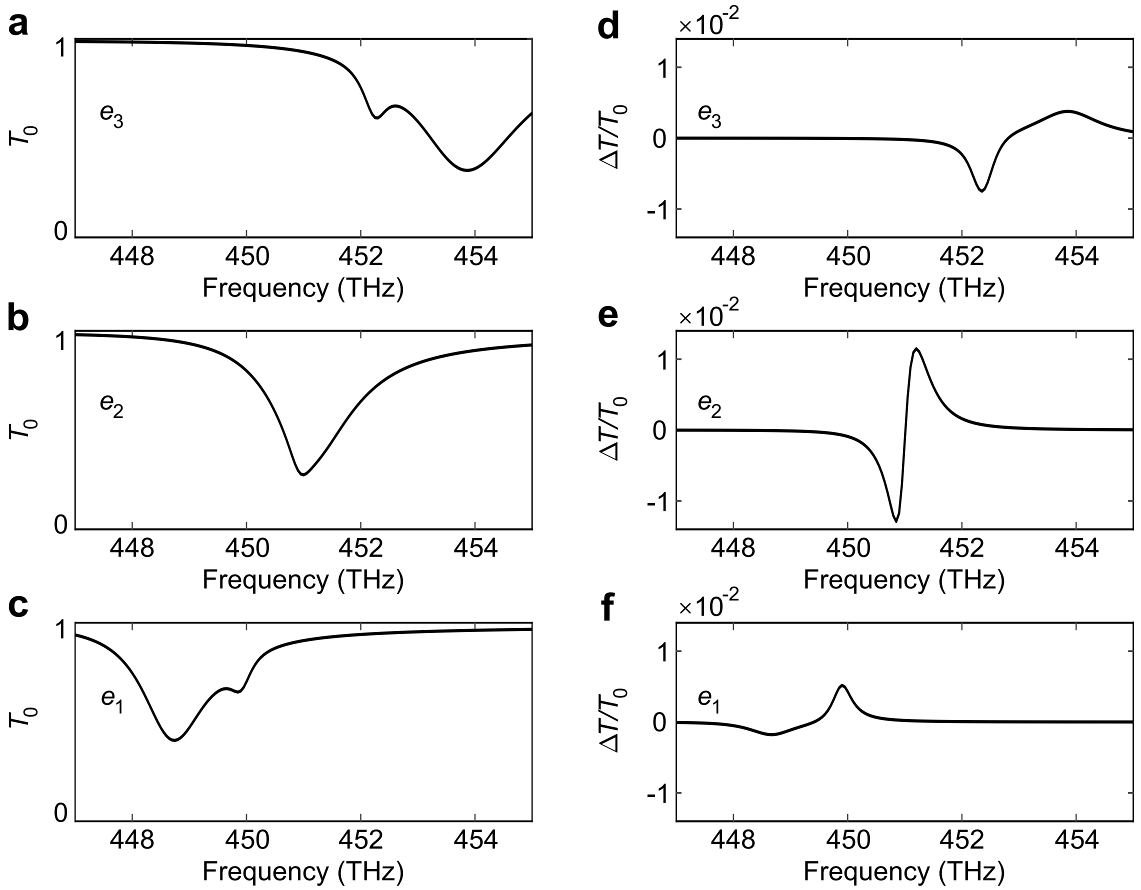

Figure S3: TMS transmittance and normalized differential transmittance. (a) T_0 of the bare TMS for LCP light for eccentricity $e_3 \approx 0.86$. (b) T_0 of the bare TMS for LCP light for eccentricity $e_2 \approx 0.85$. (c) T_0 of the bare TMS for LCP light for eccentricity $e_1 \approx 0.84$. (d) $\Delta T/T_0$ for eccentricity $e_3 \approx 0.86$. (e) $\Delta T/T_0$ for eccentricity $e_2 \approx 0.85$. (f) $\Delta T/T_0$ for eccentricity $e_1 \approx 0.84$.

Amount of chiral analyte

In this work, we place the chiral analyte in the TMS voids only, but it is an open question: How much sample one should use? On the one hand, it is in our interest to put enough chiral matter to access all near fields excited by the nanoresonator. On the other hand, the excess of the analyte eventually leads to higher chiroptical response, which is determined rather by the amount of the sample than the resonant enhancement of light-matter interaction.

To define an optimal configuration, we examine ΔT dependence on thickness L_κ of the chiral analyte placed above the TMS, meanwhile the voids are always filled with the sample. To understand the behavior of the near fields generated by the TMS, we plot the electric field intensity $|\mathbf{E}|^2$ as a function of z coordinate in Fig. S4(a). An exponential fit, which is applied to $|\mathbf{E}|^2$ in the top region, perfectly describes its decay in space. The field intensity experiences a characteristic drop $|\mathbf{E}|^2 = |\mathbf{E}|^2_{\max}/\mathrm{e}$, where e is the Euler number, at a height equal to 75 nm above the top surface, which is highlighted by the dashed line. The near fields are visualized in Fig. S4(b), where the level corresponding to 75 nm above the TMS is also indicated by the white dashed line. In Fig. S4(c), the chiroptical response for different L_κ varied from 0 to 100 nm with a step equal to $\Delta L_\kappa = 20$ nm is shown. Further, we introduce a new quantity $\Delta(\Delta T) = \Delta T(L_\kappa) - \Delta T(0)$, where $\Delta T(L_\kappa)$ and $\Delta T(0)$ are peak-to-peak differential transmittance amplitudes for the given L_κ and $L_\kappa = 0$, respectively. $\Delta(\Delta T)$ basically shows the differential transmittance increment with the increase of L_κ and evidently depends on the latter, as demonstrated in Fig. S4(d). The representation of this dependence in double-logarithmic scale in Fig. S4(e) allows us to distinguish two regions, where the data points can be fit by linear functions with different slopes. Notably, the switch between these two appears near to $L_\kappa = 75$ nm, which corresponds to the near field intensity drop in e times in the vicinity of the metasurface. Therefore, we can distinguish two regimes of ΔT enhancement: The first one (red solid line) is guaranteed by the metasurface near fields and stays dominant until the intensity of the latter experiences an exponential drop. The second one (black solid line) is rather defined by straightforward increase of the chiral

matter and is not affected by the resonant interaction with the TMS.

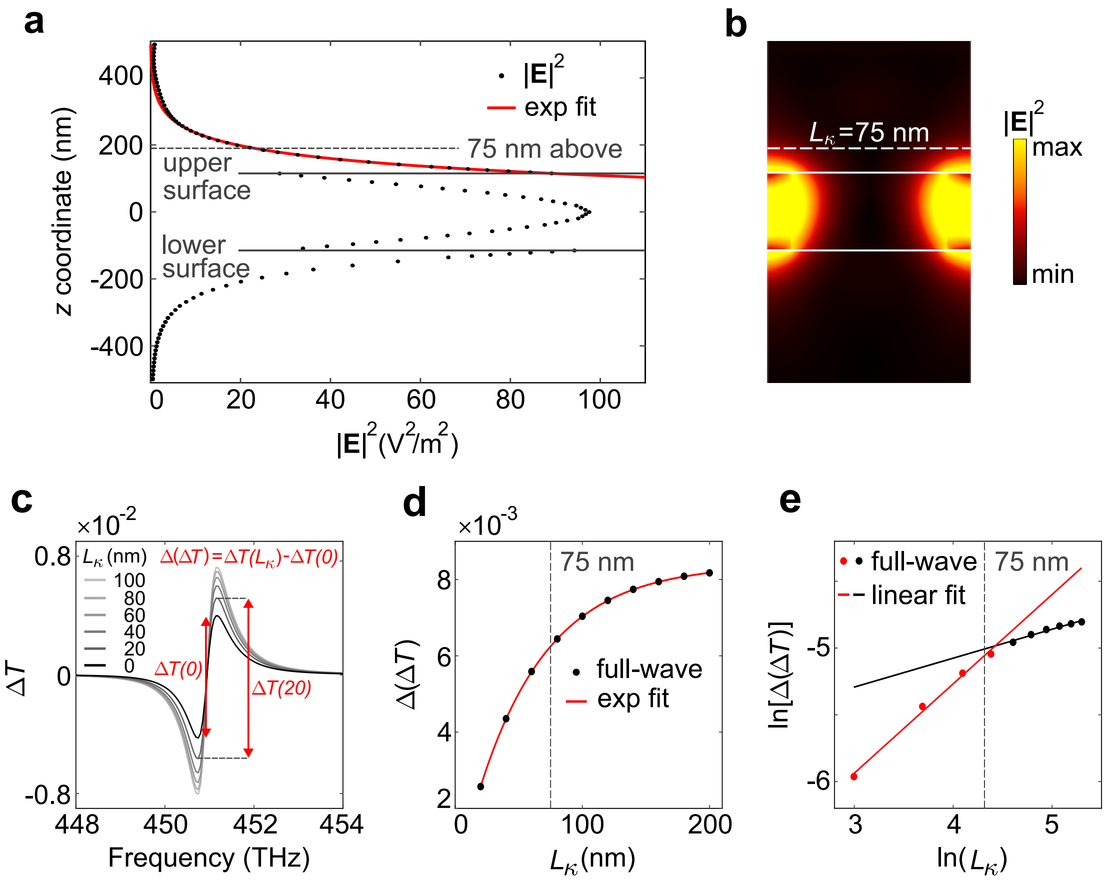

Figure S4: (a) Intensity of the near fields $|\mathbf{E}|^2$ generated by the TMS as a function of z coordinate in the vicinity of the metasurface (black dots). Solid black lines indicate the upper and lower TMS boundaries. The exponential fit applied to $|\mathbf{E}|^2$ in the top space (red solid line), and the exponential drop $|\mathbf{E}|^2 = |\mathbf{E}|^2_{\max}/\mathrm{e}$ appears at a height equal to 75 nm above the TMS (dashed gray line). (b) $|\mathbf{E}|^2$ distribution in the vicinity of the TMS in the yz plane. (c) ΔT obtained for different thickness L_κ of the chiral analyte above the TMS. The quantity $\Delta(\Delta T)$ is introduced to define the increment of ΔT with the increase of L_κ. (d) $\Delta(\Delta T)$ as a function of L_κ. (e) $\Delta(\Delta T)$ as a function of L_κ in a double-logarithmic scale. Two regimes are identified: The first one that is dominated by the TMS near fields (red dots) and the second one that is defined by the increase of the chiral analyte amount (black dots).

References

(1) Lindell, I. V.; Sihvola, A.; Tretyakov, S.; Viitanen, A. J. *Electromagnetic waves in chiral and bi-isotropic media*; Artech House Boston, 1994.

(2) Graf, F.; Feis, J.; Garcia-Santiago, X.; Wegener, M.; Rockstuhl, C.; Fernandez-Corbaton, I. Achiral, helicity preserving, and resonant structures for enhanced sensing of chiral molecules. *ACS Photonics* **2019**, *6*, 482–491.

(3) Lee, S.; Kang, J.-H.; Yoo, S.; Park, Q.-H. Robust numerical evaluation of circular dichroism from chiral medium/nanostructure coupled systems using the finite-element method. *Scientific Reports* **2018**, *8*, 8406.

(4) García-Guirado, J.; Svedendahl, M.; Puigdollers, J.; Quidant, R. Enhanced Chiral Sensing with Dielectric Nanoresonators. *Nano Letters* **2020**, *20*, 585–591.

(5) Both, S.; Schäferling, M.; Sterl, F.; Muljarov, E. A.; Giessen, H.; Weiss, T. Nanophotonic chiral sensing: How does it actually work? *ACS nano* **2022**, *16*, 2822–2832.

(6) Nesterov, M. L.; Yin, X.; Schäferling, M.; Giessen, H.; Weiss, T. The role of plasmon-generated near fields for enhanced circular dichroism spectroscopy. *Acs Photonics* **2016**, *3*, 578–583.

(7) Droulias, S. Chiral sensing with achiral isotropic metasurfaces. *Physical Review B* **2020**, *102*, 075119.

(8) Schäferling, M. *Chiral Nanophotonics: Chiral Optical Properties of Plasmonic Systems*; Springer Series in Optical Sciences; Springer International Publishing: Cham, 2017; Vol. 205.

(9) Bai, B.; Ventola, K.; Tervo, J.; Zhang, Y. Determination of the eigenpolarizations in arbitrary diffraction orders of planar periodic structures under arbitrary incidence. *Physical Review A* **2012**, *85*, 053808.

(10) Yoo, S.; Park, Q.-H. Metamaterials and chiral sensing: a review of fundamentals and applications. *Nanophotonics* **2019**, *8*, 249–261.

(11) Barron, L. D. *Molecular light scattering and optical activity*; Cambridge University Press, 2009.
